# High-quality permanent draft genome sequence of *Ensifer meliloti* strain 4H41, an effective salt- and drought-tolerant microsymbiont of *Phaseolus vulgaris*

**DOI:** 10.1186/s40793-015-0005-1

**Published:** 2015-07-02

**Authors:** Ridha Mhamdi, Julie Ardley, Rui Tian, Rekha Seshadri, T.B.K. Reddy, Amrita Pati, Tanja Woyke, Victor Markowitz, Natalia Ivanova, Nikos Kyrpides, Wayne Reeve

**Affiliations:** 1 Laboratory of Legumes, Centre of Biotechnology of Borj-Cedria, BP 901, Hammam-Lif, 2050 Tunisia; 2 Centre for Rhizobium Studies, Murdoch University, Murdoch, WA Australia; 3 DOE Joint Genome Institute, Walnut Creek, CA USA; 4 Biological Data Management and Technology Center, Lawrence Berkeley National Laboratory, Berkeley, CA USA; 5 Department of Biological Sciences, Faculty of Science, King Abdulaziz University, Jeddah, Saudi Arabia

**Keywords:** Root-nodule bacteria, Nitrogen fixation, *Alphaproteobacteria*, *Ensifer*, *Phaseolus vulgaris*

## Abstract

*Ensifer meliloti* 4H41 is an aerobic, motile, Gram-negative, non-spore-forming rod that can exist as a soil saprophyte or as a legume microsymbiont of common bean (*Phaseolus vulgaris*). Strain 4H41 was isolated in 2002 from root nodules of *P. vulgaris* grown in South Tunisia from the oasis of Rjim-Maatoug. Strain 4H41 is salt- and drought-tolerant and highly effective at fixing nitrogen with *P. vulgaris*. Here we describe the features of *E. meliloti* 4H41, together with genome sequence information and its annotation. The 6,795,637 bp high-quality permanent draft genome is arranged into 47 scaffolds of 47 contigs containing 6,350 protein-coding genes and 72 RNA-only encoding genes, and is one of the rhizobial genomes sequenced as part of the DOE Joint Genome Institute 2010 Genomic Encyclopedia for Bacteria and Archaea-Root Nodule Bacteria (GEBA-RNB) project proposal.

**Electronic supplementary material:**

The online version of this article (doi:10.1186/s40793-015-0005-1) contains supplementary material, which is available to authorized users.

## Introduction

Common bean (*Phaseolus vulgaris*) represents a very valuable source of proteins for low-income populations in Latin America and Africa [1]. However, this legume is considered to be a poor nitrogen-fixing pulse in comparison to other grain legumes [2]. This problem is generally attributed to the ineffectiveness of the native rhizobia, which is typically linked to the nodulation promiscuity of *P. vulgaris* [3-6] or to adverse abiotic conditions [7,8]. Salinity and drought are considered to be the major abiotic constraints that affect legumes in Tunisia and other countries. The selection of superior strains of rhizobia capable of assuring optimal nitrogen fixation under these adverse conditions is of high interest. Attention has therefore been directed to the isolation and characterization of rhizobial strains from various marginal areas that are subject to adverse climatic and edaphic conditions. In this context, *Ensifer meliloti* (formerly ‘*Sinorhizobium meliloti* *’*) strain 4H41 was isolated from root nodules of common bean grown in sandy, slightly alkaline soil from the oasis of Rjim-Maatoug in South Tunisia [9].

*E. meliloti* is classically considered to be a specific microsymbiont of the genera *Medicago*, *Melilotus* and *Trigonella* [10], however, recent studies have identified strains of *E. meliloti* that effectively nodulate *P. vulgaris* or several other legume species in northern Africa, South Africa and the Canary Islands [11-14]. Strain 4H41 induced nitrogen-fixing nodules on *P. vulgaris* but failed to nodulate *Medicago* spp. The phylogenetic analysis of *nifH* and *nodC* genes showed that strain 4H41 should be classified in a novel symbiovar (sv. *mediterranense*) [15]. The symbiovar *mediterranense* has also been used to describe *Ensifer fredii* and *Ensifer americanum* strains that can nodulate and fix nitrogen with *P. vulgaris*, species of Mexican *Acacia* (now reclassified as *Vachellia* and *Senegalia* [16]) and *Leucaena leucocephala* [17]. Strain 4H41 was able to grow in 4.4% NaCl (750 mM), while the *P. vulgaris* commercial inoculant *Rhizobium tropici* CIAT899^T^ did not grow in salt concentrations higher than 1.8% [9]. Inoculationwater deficiency showed that strain 4H41 was more competitive and more effective than strain experiments under CIAT899^T^ [7]. In field trials, *P. vulgaris* inoculated with strain 4H41 showed a significant increase in nodule number, shoot dry weight and grain yield even in non-irrigated fields. Under these conditions of water deficiency, nodulation by indigenous rhizobia was totally absent. However, when common bean was grown in adequately irrigated soil samples from these fields, numerous nodules could be observed, suggesting that, in contrast to 4H41, the native rhizobia were not tolerant of water deficiency [7]. Because of its effectiveness and high salt tolerance, strain 4H41 is considered to be an elite candidate for inoculant formulation in order to promote cultivation of common bean under salt and drought constraints. This strain has therefore been selected as part of the DOE Joint Genome Institute 2010 *Genomic Encyclopedia for Bacteria and Archaea-Root Nodule Bacteria* (GEBA-RNB) sequencing project [18]. Here we present a summary classification and a set of general features for *E. meliloti* strain 4H41, together with a description of its genome sequence and annotation.

## Organism information

### Classification and features

**Figure 1:**
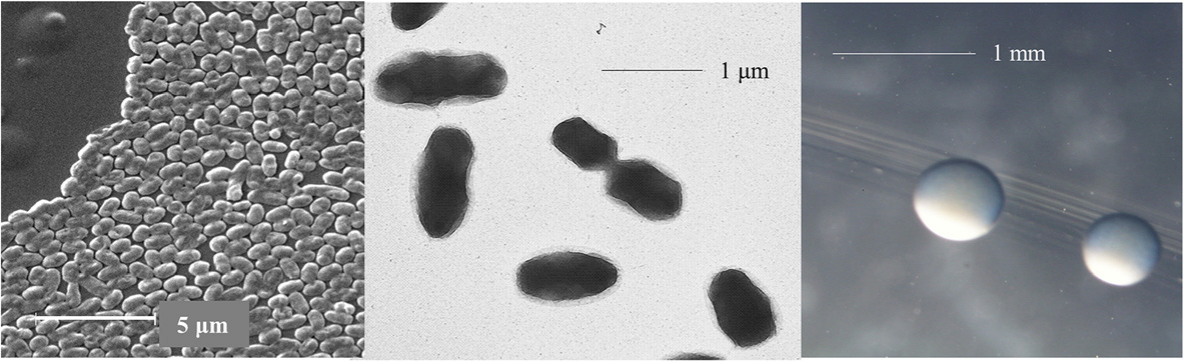
Images of *Ensifer meliloti* 4H41 using scanning (Left) and transmission (Center) electron microscopy and the appearance of colony morphology on solid media (Right).

*E. meliloti* 4H41 is a motile, Gram-negative strain in the order *Rhizobiales* of the class *Alphaproteobacteria*. The rod shaped form (Figure 1 Left and Center) has dimensions of approximately 0.25-0.5 μm in width and 0.75-1.0 μm in length. It is fast growing, forming colonies within 3–4 days when grown on half strength Lupin Agar (½LA) [19], tryptone-yeast extract agar (TY) [20] or a modified yeast-mannitol agar (YMA) [21] at 28°C. Colonies on ½LA are white-opaque, slightly domed and moderately mucoid with smooth margins (Figure 1 Right).

**Figure 2:**
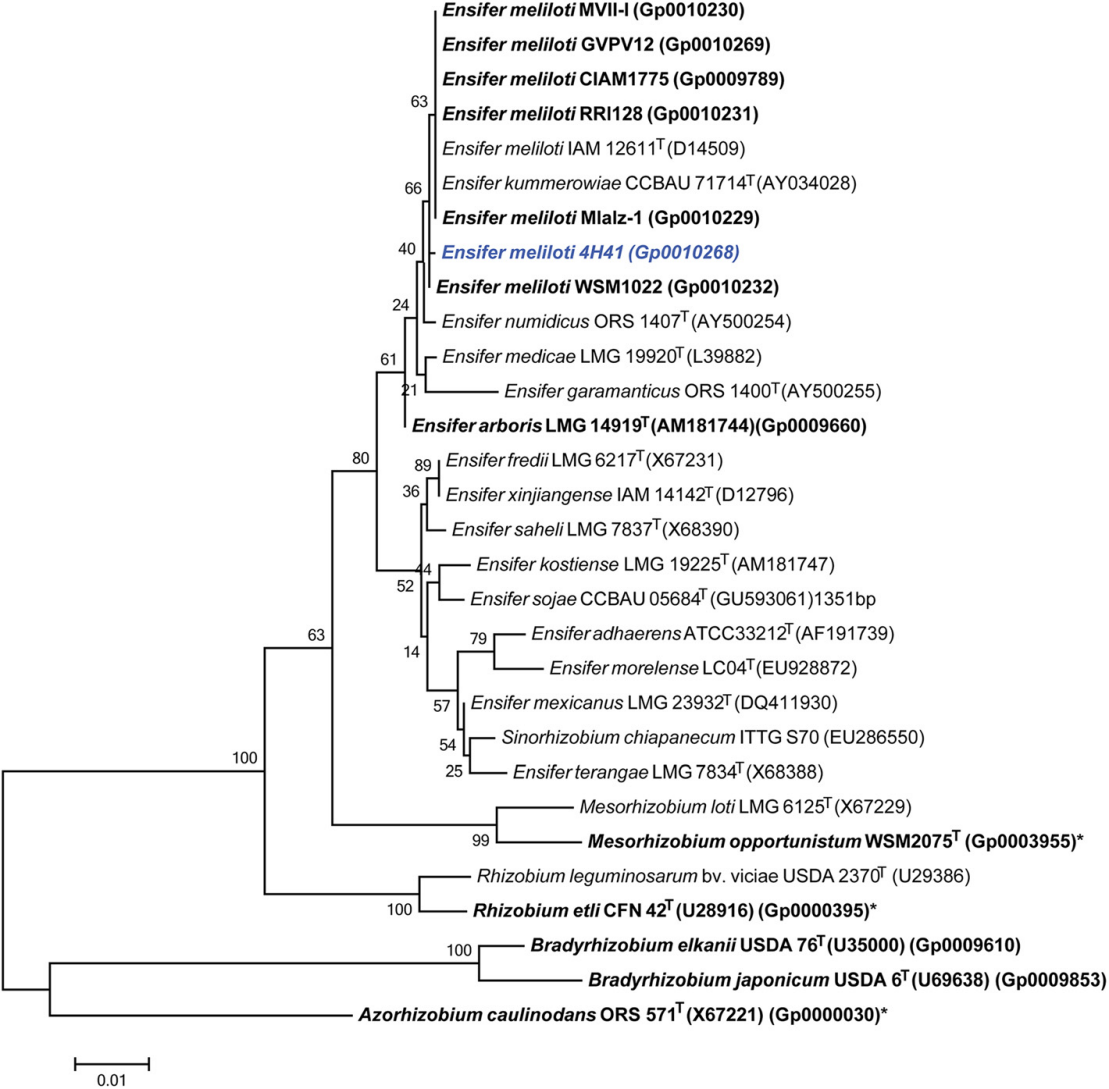
Phylogenetic tree showing the relationship of *Ensifer meliloti* 4H41 (shown in bold blue print) to *Ensifer* spp. and other root nodule bacteria species in the order *Rhizobiales*, based on aligned sequences of the 16S rRNA gene (1,240 bp internal region). (The species name “*Sinorhizobium chiapanecum*” has not been validly published.) *Azorhizobium caulinodans* ORS 571^T^ was used as an outgroup. All sites were informative and there were no gap-containing sites. Phylogenetic analyses were performed using MEGA, version 6 [45]. The tree was built using the Maximum-Likelihood method with the General Time Reversible model [46]. Bootstrap analysis [47] with 500 replicates was performed to assess the support of the clusters. Type strains are indicated with a superscript T. Strains with a genome sequencing project registered in GOLD [23] are in bold font and the GOLD ID is provided after the GenBank accession number, where this is available. Finished genomes are indicated with an asterisk.

Figure 2 shows the phylogenetic relationship of *E. meliloti* 4H41 in a 16S rRNA sequence based tree. This strain is the most similar to *Ensifer meliloti* LMG 6133^T^ and *Ensifer numidicus* ORS 1407^T^ based on the 16S rRNA gene alignment with sequence identities of 99.85% and 99.63%, respectively, as determined using the EzTaxon-e server [22]. Minimum Information about the Genome Sequence (MIGS) for 4H41 is provided in Table 1 and Additional file 1: Table S1.

**Table 1:** **Classification and general features of** ***Ensifer meliloti*** **4H41** [48,49]

**MIGS ID**	**Property**	**Term**	**Evidence code** ^**a**^
	Classification	Domain *Bacteria*	TAS [50]
		Phylum *Proteobacteria*	TAS [51,52]
		Class *Alphaproteobacteria*	TAS [51,53]
		Order *Rhizobiales*	TAS [51,54]
		Family *Rhizobiaceae*	TAS [51,55]
		Genus *Ensifer*	TAS [56,57]
		Species *Ensifer meliloti*	TAS [56]
		Strain: 4H41	
	Gram stain	Negative	IDA
	Cell shape	Rod	IDA
	Motility	Motile	IDA
	Sporulation	Non-sporulating	NAS
	Temperature range	10-40°C	TAS [56]
	Optimum temperature	28°C	TAS [56]
	pH range; Optimum	5-9.5; 6.5-8	TAS [56]
	Carbon source	Mannitol	TAS [9]
MIGS-6	Habitat	Soil; root nodule on host (*Phaseolus vulgaris*)	TAS [9]
MIGS-6.3	Salinity	0.4-4.4% (w/v)	TAS [9]
MIGS-22	Oxygen requirement	Aerobic	NAS [9]
MIGS-15	Biotic relationship	Free living, symbiotic	TAS [9]
MIGS-14	Pathogenicity	Non-pathogen	NAS
MIGS-4	Geographic location	Rjim Maatoug, Tunisia	TAS [9]
MIGS-5	Sample collection date	2002	TAS [9]
MIGS-4.1 MIGS-4.2	Longitude	7.99	TAS [9]
	Latitude	33.3245	TAS [9]
MIGS-4.3	Depth	0-10 cm	NAS
MIGS-4.4	Altitude	40 m	TAS [9]

Evidence codes – IDA: Inferred from Direct Assay; TAS: Traceable Author Statement (i.e., a direct report exists in the literature); NAS: Non-traceable Author Statement (i.e., not directly observed for the living, isolated sample, but based on a generally accepted property for the species, or anecdotal evidence). These evidence codes are from the Gene Ontology project [58,59].

### Symbiotaxonomy

*E. meliloti* strain 4H41 is highly effective for nitrogen fixation with *P. vulgaris*, but is unable to nodulate several legume species that have previously been identified as *E. meliloti* hosts [14]. The symbiotic characteristics of *E. meliloti* strain 4H41 on a range of selected phylogenetically diverse hosts are provided in Table 2.

**Table 2:** **Nodulation and N**_**2**_ **fixation properties of** ***Ensifer meliloti*** **4H41 on various hosts**

**Legume Species**	**Legume Tribe**	**Nod** ^*^	**Fix**	**Comment**
*Argyrolobium uniflorum*	Genisteae	Nod-	Fix-	
*Genista saharae*	Genisteae	Nod-	Fix-	
*Medicago ciliaris*	Trifolieae	Nod-	Fix-	
*Medicago laciniata*	Trifolieae	Nod-	Fix-	
*Medicago sativa*	Trifolieae	Nod-	Fix-	
*Medicago truncatula*	Trifolieae	Nod-	Fix-	
*Phaseolus vulgaris*	Phaseoleae	Nod^**+**^	Fix^**+**^	Highly effective
*Retama raetam*	Genisteae	Nod-	Fix-	

^*^‘+’ and ‘-’ denote presence or absence, respectively, of nodulation (Nod) or N_2_ fixation (Fix).

## Genome sequencing information

### Genome project history

This organism was selected for sequencing on the basis of its environmental and agricultural relevance to issues in global carbon cycling, alternative energy production, and biogeochemical importance, and is part of the *Genomic Encyclopedia of Bacteria and Archaea*, The Root Nodulating Bacteria chapter (GEBA-RNB) project at the U.S. Department of Energy, Joint Genome Institute (JGI). The genome project is deposited in the Genomes OnLine Database [23] and a high-quality permanent draft genome sequence is deposited in IMG [24]. Sequencing, finishing and annotation were performed by the JGI [25]. A summary of the project information is shown in Table 3.

**Table 3:** **Genome sequencing project information for** ***Ensifer meliloti*** **4H41**

**MIGS ID**	**Property**	**Term**
MIGS-31	Finishing quality	High-quality permanent draft
MIGS-28	Libraries used	Illumina Standard shotgun library
MIGS-29	Sequencing platforms	Illumina HiSeq 2000
MIGS-31.2	Fold coverage	122.2× Illumina
MIGS-30	Assemblers	Velvet version 1.1.04; Allpaths-LG version r41043
MIGS-32	Gene calling methods	Prodigal 1.4
	Locus Tag	B075 [60]
	GenBank ID	AQWP00000000
	GenBank Date of Release	Apr 20 2013
	GOLD ID	Gp0010268 [60]
	BIOPROJECT	169747
MIGS-13	Source Material Identifier	4H41, WSM4555
	Project relevance	Symbiotic N_2_ fixation, agriculture

### Growth conditions and genomic DNA preparation

*E. meliloti* 4H41 was cultured to mid logarithmic phase in 60 ml of TY rich media [26] on a gyratory shaker at 28°C. DNA was isolated from the cells using a CTAB (Cetyl trimethyl ammonium bromide) bacterial genomic DNA isolation method [27].

### Genome sequencing and assembly

The draft genome of *E. meliloti* 4H41 was generated at the DOE Joint Genome Institute (JGI) using the Illumina technology [28]. An Illumina standard shotgun library was constructed and sequenced using the Illumina HiSeq 2000 platform which generated 17,481,364 reads totaling 2,622.2 Mbp. All general aspects of library construction and sequencing performed at the JGI can be found on the JGI website [29]. All raw Illumina sequence data was passed through DUK, a filtering program developed at JGI, which removes known Illumina sequencing and library preparation artifacts [30]. The following steps were then performed for assembly: (1) filtered Illumina reads were assembled using Velvet (version 1.1.04) [31], (2) 1–3 Kbp simulated paired end reads were created from Velvet contigs using wgsim [32], (3) Illumina reads were assembled with simulated read pairs using Allpaths–LG (version r41043) [33]. Parameters for assembly steps were: 1) Velvet (velveth: 63 -shortPaired and velvetg: −very_clean yes –export-Filtered yes –min_contig_lgth 500 –scaffolding no –cov_cutoff 10) 2) wgsim (−e 0 –1 100 –2 100 –r 0 –R 0 –X 0) 3) Allpaths–LG (PrepareAllpathsInputs: PHRED_64 = 1 PLOIDY = 1 FRAG_COVERAGE = 125 JUMP_COVERAGE = 25 LONG_JUMP_COV = 50, RunAllpathsLG: THREADS = 8 RUN = std_shredpairs TARGETS = standard VAPI_WARN_ONLY = True OVERWRITE = True). The final draft assembly contained 47 contigs in 47 scaffolds. The total size of the genome is 6.8 Mbp and the final assembly is based on 830.5 Mbp of Illumina data, which provides an average 122.2x coverage of the genome.

### Genome annotation

Genes were identified using Prodigal [34] as part of the DOE-JGI genome annotation pipeline [35,36]. The predicted CDSs were translated and used to search the National Center for Biotechnology Information (NCBI) nonredundant database, UniProt, TIGRFam, Pfam, KEGG, COG, and InterPro databases. The tRNAScanSE tool [37] was used to find tRNA genes, whereas ribosomal RNA genes were found by searches against models of the ribosomal RNA genes built from SILVA [38]. Other non–coding RNAs such as the RNA components of the protein secretion complex and the RNase P were identified by searching the genome for the corresponding Rfam profiles using INFERNAL [39]. Additional gene prediction analysis and manual functional annotation was performed within the Integrated Microbial Genomes Expert Review (IMG-ER) [40] developed by the Joint Genome Institute, Walnut Creek, CA, USA.

## Genome properties

The genome is 6,795,637 nucleotides with 62.01% GC content (Table 4) and comprised of 47 scaffolds of 47 contigs. From a total of 6,422 genes, 6,350 were protein encoding and 72 RNA only encoding genes. The majority of protein-coding genes (82.01%) were assigned a putative function whilst the remaining genes were annotated as hypothetical. The distribution of genes into COGs functional categories is presented in Table 5.

**Table 4:** **Genome statistics for** ***Ensifer meliloti*** **4H41**

**Attribute**	**Value**	**% of Total**
Genome size (bp)	6,795,637	100.00
DNA coding (bp)	5,911,163	86.98
DNA G + C (bp)	4,213,729	62.01
DNA scaffolds	47	100.00
Total genes	6,422	100.00
Protein coding genes	6,350	98.88
RNA genes	72	1.12
Pseudo genes	1	0.02
Genes in biosynthetic clusters	399	6.21
Genes with function prediction	5,267	82.01
Genes assigned to COGs	4,715	73.42
Genes assigned Pfam domains	5,435	84.63
Genes with signal peptides	553	8.61
Genes with transmembrane helices	1,426	22.20
CRISPR repeats	0	-

**Table 5:** **Number of genes of** ***Ensifer*** **meliloti 4H41 associated with general COG functional categories**

**Code**	**Value**	**% of total (5,383)**	**Description**
J	219	4.07	Translation, ribosomal structure and biogenesis
A	0	0.00	RNA processing and modification
K	465	8.64	Transcription
L	134	2.49	Replication, recombination and repair
B	1	0.02	Chromatin structure and dynamics
D	39	0.72	Cell cycle control, cell division, chromosome partitioning
V	107	1.99	Defense mechanisms
T	215	3.99	Signal transduction mechanisms
M	289	5.37	Cell wall/membrane biogenesis
N	67	1.24	Cell motility
W	30	0.56	Extracellular structures
U	83	1.54	Intracellular trafficking and secretion
O	201	3.73	Posttranslational modification, protein turnover, chaperones
C	333	6.19	Energy production and conversion
G	590	10.96	Carbohydrate transport and metabolism
E	625	11.61	Amino acid transport and metabolism
F	112	2.08	Nucleotide transport and metabolism
H	243	4.51	Coenzyme transport and metabolism
I	236	4.38	Lipid transport and metabolism
P	295	5.48	Inorganic ion transport and metabolism
Q	168	3.12	Secondary metabolites biosynthesis, transport and catabolism
R	546	10.14	General function prediction only
S	337	6.26	Function unknown
X	48	0.89	Mobilome: prophages, transposons
-	1,707	26.58	Not in COGS

## Conclusion

Based on the 16S rRNA gene alignment, 4H41 is most closely related to *Ensifer meliloti* LMG 6133^T^, a *Medicago* microsymbiont [41] and *Ensifer numidicus* ORS 1407^T^, which effectively nodulates *Argyrolobium uniflorum* [42]. In contrast to these two strains, 4H41 is unable to nodulate either of these hosts. Strain 4H41 is one of 27 strains of *E. meliloti* with sequenced genomes deposited in the IMG database. Of these, 4H41 and strain GVPV12 [12] are the only two *E.* meliloti strains that have been isolated from, and are able to nodulate and fix nitrogen with, *P. vulgaris*. As the other sequenced *E.* meliloti strains are microsymbionts of *Medicago* spp., 4H41 is therefore a useful strain for comparing the molecular determinants of symbiosis in rhizobia with similar chromosomal backgrounds but which nodulate different legume hosts.

The genome size of the *E. meliloti* strains ranges from 6.6 – 8.9 Mbp; at 6.80 Mbp, the 4H41 genome is at the lower end of this range. It contains one pseudo gene, the numbers of which are highly variable in the sequenced *E. meliloti* genomes and can be up to 444 (*E.* melilotiAK83). In common with the other *E. meliloti* genomes, 4H41 possesses a large number of genes assigned to COG functional categories for transport and metabolism of amino acids (12.22%), carbohydrates (11.03%), inorganic ions (5.3%), lipids (3.97%) and coenzymes (3.59%), and involved in transcription (8.78%), and signal transduction (3.58%). Genome analysis has revealed three distinct *nodA* genes, two coding for NodA proteins composed of 196 amino acids while the third encodes a NodA protein of 141 amino acids that lacks a 55 amino acid segment at the C-terminus. All three *nodA* copies are harboured within a symbiotic region of the genome and have highest sequence identity at the protein level with the common bean-nodulating strains *Ensifer fredii* GR64 [43] and *E. meliloti* GVPV12. Three distinct *nodA* genes are also found in the *P. vulgaris* commercial inoculant strains *Rhizobium tropici* CIAT 899 and *Rhizobium* sp. PRF 81 [44].

4H41 is salt- and drought-tolerant and highly effective for nitrogen fixation with *P. vulgaris*, and as such is a valuable inoculant strain. Analysis of its sequenced genome and comparison with the genomes of other sequenced *E. meliloti* and with RNB that nodulate the common bean will yield new insights into the molecular basis of salt- and drought-tolerance in rhizobia and into the molecular determinants of symbiotic specificity and nitrogen fixation in the important pulse legume *P. vulgaris*.

## Additional file

Additional file 1: Table S1. Associated MIGS record.

## Abbreviations

GEBA-RNB: Genomic Encyclopedia for Bacteria and Archaea-Root Nodule Bacteria
