# Supplementary material for: High-quality permanent draft genome sequence of Ensifer meliloti strain 4H41, an effective salt- and drought-tolerant microsymbiont of Phaseolus vulgaris
Source: Stand Genomic Sci. 2015 Jul 2;10:34. doi: 10.1186/s40793-015-0005-1 (PMC4571125; doi:10.1186/s40793-015-0005-1)
Supplement: Additional file 1: Table S1. — Associated MIGS record. [file 40793_2015_5_MOESM1_ESM.docx]

Additional file 1: Associated MIGS record

**Table S1.** Associated MIGS record for 4H41

| **MIGS-ID** | field name | description |
| --- | --- | --- |
| **MIGS-1** | Submit to INSDC/Trace archives |  |
| **1.1** | PID | Gp0010268 |
| **1.2** | Trace Archive |  |
| **MIGS-2** | MIGS CHECK LIST TYPE |  |
| **MIGS-3** | Project Name | GEBA - Root Nodulating Bacteria |
| **MIGS-4** | Geographic Location | Rjim Maatoug, Tunisia |
| **4.1** | Latitude | 33.3245 |
| **4.2** | Longitude | 7.99 |
| **4.3** | Depth | 1-10 cm |
| **4.4** | Altitude | 40 m |
| **MIGS-5** | Time of Sample collection | 2002 |
| **MIGS-6** | Habitat (EnvO) | Soil, root nodule, legume host |
| **6.1** | temperature | 28 |
| **6.2** | pH | 6.5-8 |
| **6.3** | salinity | 0.4-4.4% (w/v) |
| **6.4** | chlorophyll |  |
| **6.5** | conductivity |  |
|  |  |  |
| **6.6** | light intensity |  |
| **6.7** | dissolved organic carbon (DOC) |  |
| **6.8** | current |  |
| **6.9** | atmospheric data |  |
| **6.10** | density |  |
| **6.11** | alkalinity |  |
| **6.12** | dissolved oxygen |  |
| **6.13** | particulate organic carbon (POC) |  |
| **6.14** | phosphate |  |
| **6.15** | nitrate |  |
| **6.16** | sulfates |  |
| **6.17** | sulfides |  |
| **6.18** | primary production |  |
| **MIGS-7** | Subspecific genetic lineage | [*Ensifer meliloti*](http://dx.doi.org/10.1601/nm.1335) 4H41 |
| **MIGS-9** | Number of replicons |  |
| **MIGS-10** | Extrachromosomal elements |  |
| **MIGS-11** | Estimated Size | 6,795,637 bp |
| **MIGS-12** | Reference for biomaterial or Genome report |  |
| **MIGS-13** | Source material identifiers | 4H41 |
| **MIGS-14** | Known Pathogenicity | Non-pathogen |
|  |  |  |
| **MIGS-15** | Biotic Relationship | Symbiotic |
| **MIGS-16** | Specific Host | [*Phaseolus vulgaris*](http://www.theplantlist.org/tpl1.1/record/ild-2934) |
| **MIGS-17** | Host specificity or range (taxid) | [*Phaseolus vulgaris*](http://www.theplantlist.org/tpl1.1/record/ild-2934) |
| **MIGS-18** | Health status of Host | Healthy; effective nitrogen fixation |
| **MIGS-19** | Trophic Level |  |
| **MIGS-22** | Relationship to Oxygen | Aerobe |
| **MIGS-23** | Isolation and Growth conditions | TY medium , 28°C, aerobe |
| **MIGS-27** | Nucleic acid preparation | CTAB |
| **MIGS-28** | Library construction | Illumina standard PE |
| **28.1** | Library size | 2,622.2 Mbp |
| **28.2** | Number of reads | 17,481,364 |
| **28.3** | vector |  |
| **MIGS-29** | Sequencing method | Illumina HiSeq 2000 platform |
| **MIGS-30** | Assembly |  |
| **30.1** | Assembly method | Velvet, version 1.1.04, ALLPATHS r41043 |
| **30.2** | estimated error rate |  |
| **30.3** | method of calculation |  |
| **MIGS-31** | Finishing strategy |  |
| **31.1** | Status | High-quality permanent draft |
| **31.2** | coverage | 122.2x |
| **31.3** | contigs | 47 |
| **MIGS-32** | Relevant SOPs |  |
| **MIGS-33** | Relevant e-resources |  |
